# Nox4 Expression Is Not Required for OVX‐Induced Osteoblast Senescence and Bone Loss in Mice

**DOI:** 10.1002/jbm4.10376

**Published:** 2020-07-23

**Authors:** Jin‐Ran Chen, Oxana P Lazarenko, Haijun Zhao, Umesh D Wankhade, Kim Pedersen, James Watt, Martin J J Ronis

**Affiliations:** ^1^ Department of Pediatrics University of Arkansas for Medical Sciences Little Rock AR USA; ^2^ Arkansas Children's Nutrition Center Little Rock AR USA; ^3^ Department of Pharmacology and Experimental Therapeutics Louisiana State University Health Sciences Center New Orleans LA USA

**Keywords:** NOX4, OSTEOBLAST, SENESCENCE, SEX STEROID DEFICIENCY

## Abstract

Estrogen deficiency and aging play critical roles in the pathophysiology of bone as a result of increased oxidative stress. It has been suggested that prevention of NADPH oxidase‐ (Nox‐) dependent accumulation of ROS may be an approach to potentially minimize bone loss caused by these conditions. Using ovariectomized (OVX) and Nox4 gene‐deletion mouse models, we investigated the role of Nox4 in OVX‐induced bone loss and osteoblast senescence signaling. Six‐month‐old WT C57Bl6 mice were allocated to a sham control group, OVX, and OVX plus E2 treatment group for 8 weeks. Decreased bone mass including BMD and BMC were found in the OVX group compared with the sham control (*p* < 0.05); E2 treatment completely reversed OVX‐induced bone loss. Interestingly, the prevention of OVX‐induced bone loss by E2 was associated with the elimination of increased senescence signaling in bone osteoblastic cells from the OVX group. E2 blunted OVX‐induced p53 and p21 overexpression, but not p16 and Nox4 in bone. In addition, 8‐ and 11‐month‐old Nox4 KO female mice were OVX for 8 weeks. Significant bone loss and increased bone osteoblastic cell senescence signaling occurred not only in Nox4 KO OVX mice compared with sham‐operated animals, but also in 11‐month‐old Nox4 KO sham mice compared with 8‐month‐old Nox4 KO sham mice (*p* < 0.05). These data suggest that Nox4‐mediated ROS in bone osteoblastic cells may be dispensable for sex steroid deficiency‐induced bone loss and senescence. © 2020 The Authors. *JBMR Plus* published by Wiley Periodicals, Inc. on behalf of American Society for Bone and Mineral Research.

## Introduction

Bone turnover and bone mass dramatically undergo sex steroid and age‐dependent regulation. As a result, in both humans and rodent animal models, bone degeneration occurs because of sex steroid deficiency or aging. Although the mechanisms of sex steroid deficiency‐ or aging‐induced bone loss may be slightly different, bone degeneration in both conditions is a process in which osteoblast bone formation and osteoclast bone resorption are uncoupled.^(^ ^1^ ^)^ Acceptable explanations for the uncoupling bone remodeling include increased oxidative stress or accumulated intracellular ROS,^(^ ^2^ ^)^ increased osteoclast activity,^(^ ^3^ ^)^ and increased osteoblast apoptosis.^(^ ^4^ ^)^ Based on results from previous research, therapeutics for the treatment of degenerative bone loss usually are antiresorptives such as bisphosphonates,^(^ ^5^ ^)^ bone anabolic agents such as PTH and PTHrP,^(^ ^6^ ^)^ and dietary antioxidants such as vitamins C and E.^(^ ^7^ ^)^ However, for many years, using these compounds for the treatment of degenerative bone disorders has only yielded suboptimal results.^(^ ^8^ ^)^ In addition to previous mechanistic studies on sex steroid deficiency or aging‐induced bone loss, we hypothesized that degenerative bone loss may also be caused by increased osteoblast senescence pathways.^(^ ^9^ ^)^ Indeed, in many cases, the actual osteoblast numbers are not reduced in ovariectomized (OVX) animal models.^(^ ^10^ ^)^ Diminished osteoblast function might be a crucial factor leading to bone loss. We have previously shown in other animal models that chronic alcohol‐induced bone loss was significantly associated with oxidative stress‐mediated osteoblast senescence.^(^ ^9^ ^)^

In adults, bone marrow stromal cells and periosteal osteoblast precursors are thought to be two major potential sources of new functional osteoblasts.^(^ ^11^ ^)^ After functional osteoblasts are differentiated, it is estimated that these osteoblasts have a lifetime of approximately 10 days,^(^ ^12^ ^)^ after which these cells are thought to be lost via apoptosis, become trapped in bone matrix giving rise to osteocytes, become inactive lining cells, or perhaps become senescent and nonfunctional. Cellular senescence is generally a process of antiproliferation: It is thought to occur in a proportion of cells in many tissues during aging and most notably within tumors.^(^ ^13^, ^14^, ^15^ ^)^ In noncancer somatic cells, how cell senescence signaling plays a pathophysiologic role is not well‐understood. Moreover, how a senescent cell can be removed from a specific tissue is currently unknown. Among many senescence mediators, tumor suppressor genes such as p53, p21, and p16 have been characterized—and are critical for—the induction of senescence in most rodent cells. Another feature of senescent cells is an increase in the expression and secretion of numerous cytokines, chemokines, matrix metalloproteinases, and other proteins that can alter the local tissue environment. This has been termed the senescence‐associated secretory phenotype.^(^ ^16^, ^17^ ^)^ Senescence‐associated‐β‐galactosidase (SAβG) activity measurement may be the most useful approach to evaluate senescence status both in vitro and in vivo. Recent studies have also indicated that increased oxidative stress is significantly associated with increased senescence signaling in many cells and tissues.^(^ ^18^, ^19^, ^20^ ^)^

In animal models, it has been suggested that bone loss after ovariectomy or during aging is largely associated with decreases in bone formation rate.^(^ ^21^ ^)^ The ovariectomy‐ or age‐dependent loss of bone mass is also accompanied by substantial increases in the generation or accumulation of ROS, therefore increased oxidative stress.^(^ ^22^ ^)^ ROS can be generated by several sources; the tightly controlled and cell‐specific NADPH oxidases (Nox) represent one of the major sources of ROS signaling molecules, including superoxide, hydrogen peroxide, and hydroxyl radicals in many cell types. We have previously identified that osteoblastic cells do not express Nox3, but do express Nox1, 2, and 4, with isoforms 4 and 2 being relatively abundant.^(^ ^23^ ^)^ We have characterized the role of Nox2 in bone cells during bone development by using a mouse model in which its cofactor p47^phox^ was knocked out.^(^ ^24^ ^)^ In contrast, Nox4 has never been examined to determine whether it plays a role in osteoblast senescence signaling and bone loss in OVX or relatively older mouse models.

## Materials and Methods

### Animals

WT C57BL/6 and NADPH oxidase 4 C57BL/6J B6.129‐Nox4^tm1Kkr^/J (Nox4^−/−^) mice were obtained from Jackson Labs (Bar Harbor, ME, USA). Two‐month‐old female Nox4^−/−^ mice were crossed with male Nox4^−/−^ mice, and their offspring were used for the experiments. Dams and litters were housed in polycarbonate cages in an Association of Laboratory Animal Care‐approved animal facility at Louisiana State University Health Sciences Center–New Orleans (LSUHSC‐NO) in an environmentally controlled room at 22°C with a 12‐hour light/dark cycle, and fed standard rodent chow *ad libitum* throughout the experimental period, including pregnancy and lactation. Mice were fed chow *ad libitum* as previously described^(^ ^25^ ^)^ for up to 11 months. Body weights were recorded on a weekly basis and food intake daily for 7 days after 4 weeks on the diet. 17β‐estradiol (E2; 20 μg/kg/day) was administered using ALZET osmotic minipumps.^(^ ^23^ ^)^ Ovariectomy surgery was performed on female mice after they were anesthetized with isoflurane (2.5% isoflurane induction with 1.5 L/min oxygen, followed by 1.75% to 2.5% isoflurane maintenance with 1.0 L/min oxygen). The ovary was gently pulled out of the incision, and the ovary and fat pad were gently pulled off of the uterine horn using fine forceps. In sham‐operated animals, the ovary was pulled out through the incision, and then placed back inside the mouse. The incision was closed with wound clips, which were removed 7 to 10 days postoperatively. After the mice were sacrificed 8 weeks later, serum, legs, and vertebras were collected and stored at −80°C until use. All animal experiments were conducted under protocols approved by the LSUHSC‐NO Institutional Animal Care and Use Committee.

### Bone analyses

pQCT was performed on formalin‐fixed left tibia for bone mass–BMD measurement using a method established in our laboratory.^(^ ^26^ ^)^ A STRATEC XCT 960 M unit (XCT Research SA, Norland Medical Systems, Fort Atkins, WI, USA) specifically configured for small bone specimens was utilized. Software version 5.4 was used with thresholds of 570 mg/cm^3^ to distinguish cortical bone and 214 mg/cm^3^ to distinguish trabecular from cortical and subcortical bone. Tibial BMD and BMC were calculated. The position for pQCT scanning was defined as a distance from the proximal tibia of 1 mm below the growth plate, corresponding to 7% of the total length of the tibia. Distance between each scan was 0.5 mm; five scans (five slices) were carried out. Data were expressed as the mean of three contiguous slices with the greatest trabecular bone density.

At sacrifice, the whole‐spine bone was removed, and the L4 to L5 vertebra was fixed. Sequential dehydration was carried out using different concentrations of alcohol. L4 to L5 vertebra samples were embedded, cut, and senescence‐associated β‐galactosidase (SAβG) stained by standard histology special procedures.^(^ ^26^, ^27^ ^)^

μCT measurements of trabecular of the tibial bone after above pQCT process were evaluated by using SkyScan μCT scanner (recently upgraded SkyScan 1272; Bruker, Kontich, Belgium) at 6‐μm isotropic voxel size with an X‐ray source power of 55 kV and 145 μA, and an integration time of 300 ms. The gray‐scale images were processed by using a low‐pass Gaussian filter (σ = 0.8; support = 1) to remove noise, and a fixed threshold of 220 was used to extract the mineralized bone from the soft tissue and marrow phase. Cancellous bone was separated from the cortical regions by semiautomatically drawn contours. One‐hundred twenty slices, starting from about 1‐mm distal to the growth plate and constituting 0.70‐mm length, were evaluated for trabecular bone structure by using software provided by SkyScan (Bruker).

### Serum bone turnover markers

The serum bone formation marker, alkaline phosphatase (ALP) and the serum bone resorption marker, CTX‐1 procollagen cross‐links RatLaps were measured by Rat‐MID ALP ELISA and RatLaps ELISA, respectively, from Nordic Biosciences Diagnostic (Herlev, Denmark). A serum tartrate‐resistant acid phosphatase 5b (TRAP5b) measurement kit was purchased from the Quidel TECOmedical Group (San Diego, CA, USA). The TRAP5b assay is a two‐step direct‐capture enzyme immunoassay according to the protocol provided by manufacturer.

### Real‐time RT‐PCR analysis

Mouse L2 to L3 vertebral bone RNA were extracted using TRI Reagent (MRC Inc., Cincinnati, OH, USA) according to the manufacturer's recommendation, followed by DNase digestion and column clean‐up using QIAGEN mini columns (QIAGEN, Valencia, CA, USA). Briefly, RNA isolation from bone tissue was undertaken at the time of sacrifice. The L2 to L3 vertebra was taken, and bone marrow cells were flushed out with Eagle's MEM + Hanks' salts after cleaning the surrounding connective tissue. The L2 to L3 vertebral bone was placed in 1000‐μL TRI reagent with five metal beads and homogenized using a polytron aggregate (Kinematica AG, Luzern, Switzerland). Then 100 μL of 1‐bromo‐3‐chloropropane were added, and the mixture was centrifuged for 15 min at a speed of 16,000 rpm and a temperature of 4°C. Following this, 450‐μL supernatant was taken and an equal volume of isopropanol was added, and then centrifuged for an additional 15 min (16,000 rpm, 4°C). After washing the RNA pellet with 75% ethanol, isolated RNA was resuspended in RNAse‐free water. Reverse transcription was carried out using an iScript cDNA synthesis kit from Bio‐Rad (Hercules, CA, USA). Real‐time RT‐PCR was carried out using SYBR Green and an ABI 7500 fast‐sequence detection system (Applied Biosystems, Foster City, CA, USA). Primer for p53: F, GGA GAC ATT TTC AGG CTT ATG GA; R, GCC TTC AAA AAA CTC CTC AAC ATC; primer for p21: F, CCT TCC TCA CCT GTG TCG TCT T; R, TGG GAT GCA CTG GGT GTT CT; primer for Nox4: F, AGATTTGCCTGGAAGAACCCA; R, TCGGTAAAGTCTCTCCGCACA.

### Western blotting

L2 to L3 vertebral bone tissue proteins were extracted using a cell lysate buffer as described previously.^(^ ^28^ ^)^ p53, p21, p16, collagen 1, NFATc1, cathepsin K, and Nox4 protein expression in bone tissue were assessed by standard Western immunoblotting using antibodies recognizing these proteins, followed by incubation with secondary antibodies conjugated with horseradish peroxidase. Anti‐p53, Ms, monoclonal, #ab28 (Abcam, Cambridge, UK); anti‐p21, Rb, monoclonal, #ab109199 (Abcam); anti‐p16, Rb, monoclonal, #ab108349 (Abcam); anticollagen 1 (Col‐1a), Ms, monoclonal, #MA1‐26771 (Thermo Fisher Scientific, Waltham, MA, USA); anti‐NFATc1, Ms, monoclonal, #sc‐7294 (Santa Cruz Biotechnology, Santa Cruz, CA, USA); anticathepsin K, Rb, polyclonal, #ab19027 (Abcam); anti‐Nox4, Rb, polyclonal, #ABC459 (Millipore, Billerica, MA, USA); and anti‐βActin, Ms, monoclonal, #A1978 (Sigma‐Aldrich, St. Louis, MO, USA). β‐actin protein in bone tissue was analyzed by immunoblotting, using mouse monoclonal antibody recognizing β‐actin (Sigma‐Aldrich), followed by incubation with a secondary antimouse antibody conjugated with horseradish peroxidase (Santa Cruz Biotechnology). Immunoblots were visualized using SuperSignal West Pico chemiluminescent (Pierce, Rockford, IL, USA). Quantitation of the intensity of the bands in the autoradiograms was performed using a VersaDoc imaging system (Bio‐Rad).

### Senescence‐associated β‐galactosidase (SAβG) staining and activity assay and triple staining with Col‐1a and osteocalcin antibodies

SAβG activity assay was performed by β‐galactosidase enzyme assay kit (Promega, San Luis Obispo, CA, USA); the absorbance at 420 nm was measured according to the manufacturer's instructions. Bone tissue β‐galactosidase staining was also performed according to a method published previously.^(^ ^29^, ^30^ ^)^ Senescent cells were identified as blue‐stained cells by standard light microscopy. After SAβG staining, slides were further stained with Col‐1a and osteocalcin (antiosteocalcin, Ms, monoclonal, #ab13418; Abcam) using standard immunostaining.

### RNA‐seq‐based gene expression analysis

RNA‐seq was performed using RNA isolated from L2 to L3 vertebral bone from all groups of mice. Total RNA was isolated from L2 to L3 vertebral bone of individual mice. Poly‐A mRNA from each individual RNA sample (1 μg) was isolated using mRNA direct reagents. Equal amounts of polyA‐mRNA from two to three mice were pooled to generate three biologically distinct replicates per treatment group, representing all animals. Directional RNA‐seq libraries were prepared using NEBNext reagents (New England BioLabs, Ipswich, MA, USA) as described previously,^(^ ^31^ ^)^ validated using electrophoresis, and quantified using Qubit dsDNA reagents. Single‐read 75‐bp sequencing of libraries was performed using a NextSeq500 (Illumina, San Diego, CA, USA). Data analysis was performed via alignment of high‐quality reads using Bowtie2, and quantitation of read counts to genes using SeqMonk.^(^ ^32^, ^33^ ^)^ Differentially expressed genes were identified via DeSeq2 package in R (±two‐fold change; FDR corrected *p* values <0.05; R Foundation for Statistical Computing, Vienna, Austria; https://www.r-project.org/). The lists of differentially expressed genes were analyzed for gene ontology (GO) biological process enrichment using the BiNGO plugin in Cytoscape (https://cytoscape.org/). To specifically examine expression differences in senescence‐associated genes, we compiled a list of genes with known functions in senescence signaling. Using the mouse genome informatics GO reference (MGI; http://www.informatics.jax.org/), we identified genes with the term “senescence” in either one of three ontologies: Biological Process, Cellular Component, or Molecular functions. This list, referred to as senescence‐related genes, was further analyzed for differential expression for a targeted analysis.

### Statistical analyses

Data were expressed as means ± SE. One‐way and two‐way ANOVAs, followed by Student–Newman–Keuls post hoc analysis, were used to compare the treatment groups. Values were considered statistically significant at *p* < 0.05.

## Results

### OVX‐induced bone loss was reversed by E2 treatment in mice

**Figure 1:**
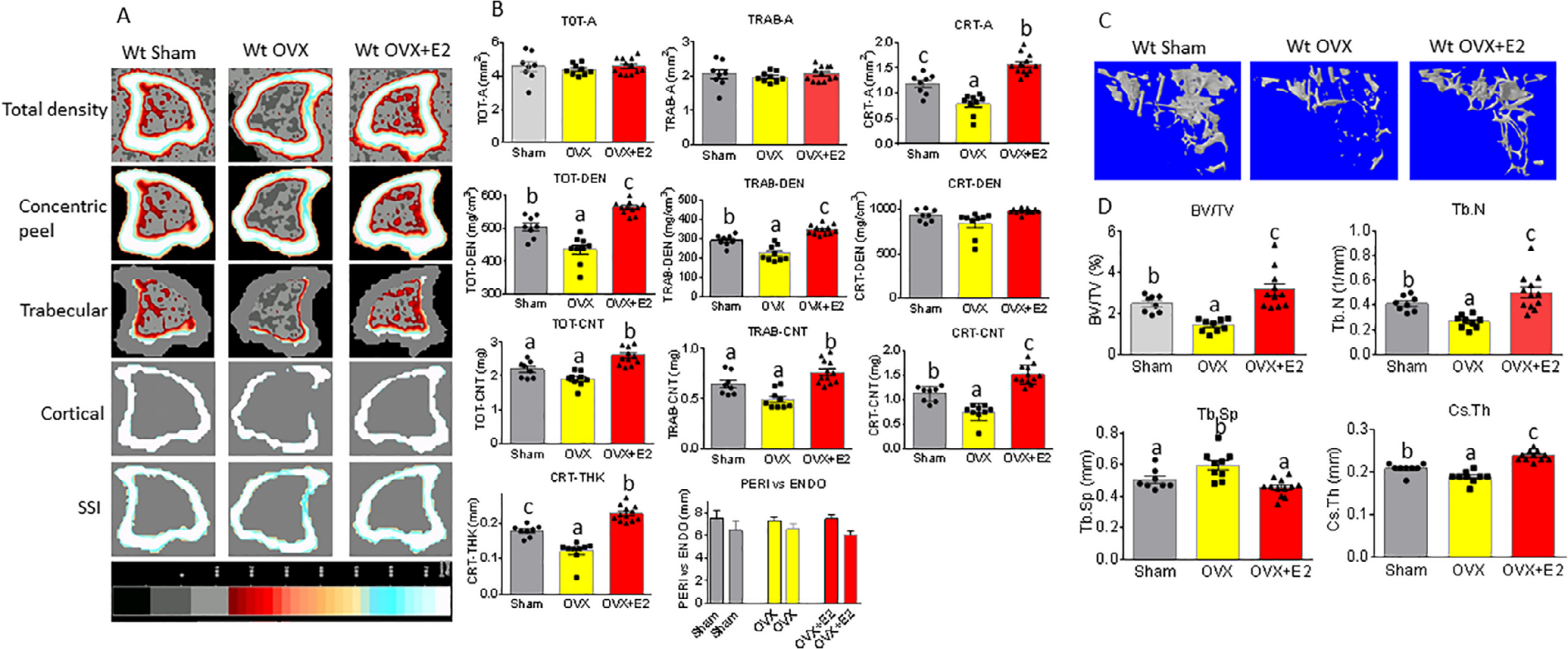
E2 reverses OVX‐induced bone loss in WT female mice. (*A*) Sagittal quantitative pQCT scan of one slice of the proximal tibia from one sample of each group of mice. Bar in the bottom shows color changes from black to white indicating bone density from low to high. (*B*) pQCT measured parameters from three mouse groups. WT sham = WT sham‐operated; WT OVX = WT ovariectomized; WT OVX + E2 = WT ovariectomized plus E2 treatment; TOT‐A, TRAB‐A, CRT‐A = total bone area, trabecular bone area, cortical bone area, mm^2^; TOT‐DEN, TRAB‐DEN, CRT‐DEN = total BMD, trabecular BMD, cortical BMD, gm/cm^3^; TOT‐CNT, TRAB‐CNT, CRT‐CNT = total BMC, trabecular BMC, cortical BMC, mg; CRT‐THK = cortical thickness, mm; PERI versus ENDO = periosteal and endosteal circumference, mm. (*C*) Representative μCT images of the proximal tibia from one sample of each group of mice. (*D*) μCT measured parameters from three mouse groups. BV/TV = bone volume / total tissue volume; Tb.N = trabecular number; Tb.Sp = trabecular separation; Cs.Th = cortical thickness. Data are expressed as mean ± SD (sham group *n* = 9; OVX group *n* = 10; OVX + E2 *n* = 11). Means with different letters differ significantly from each other, *p* < 0.05 a < b < c as determined by one‐way ANOVA followed by Student–Newman–Kules post hoc analysis for multiple pairwise comparisons.

It is known that estrogen treatment in mice is able to prevent OVX‐induced bone loss. We began our studies by analyzing changes in bone mass and structures in the proximal tibia from 6‐month‐old WT sham‐operated, 8‐week‐old OVX, and 8‐week‐old OVX plus E2‐treated female mice using two methods: pQCT and μCT. As shown in representative pQCT scans of tibial bone mass parameters in Fig. 1*A* and by their quantification in Fig. 1*B*, we first found that there were no changes in total and trabecular bone areas (Fig. 1*B*). However, the cortical bone area from OVX mice was significantly lower than that from sham mice (*p* < 0.05). E2 treatment not only prevented OVX‐induced decreases of cortical bone area, but also increased it relative to the sham group (*p* < 0.05; Fig. 1*B*). When we analyzed and compared BMD between each group, we found both the total and trabecular BMD in mice from the OVX group were significantly lower than that from the sham group (Fig. 1*B*). Interestingly, despite the significant differences in cortical area among the groups, there were no differences in cortical BMD in all three groups (Fig. 1*B*). BMC was also analyzed: Total, trabecular, and cortical BMC were all significantly lower in the OVX group compared with the sham group. The OVX plus E2 treatment group showed even higher levels of all three parameters than was observed in the sham group (*p* < 0.05; Fig. 1*B*). This was also the case for cortical thickness (Fig. 1*B*), but not for periosteal and endosteal perimeter measures (Fig. 1*B*).

Bone structures were also assessed by μCT as shown in the representative scans of below the growth plate of the tibia in Fig. 1 *C*. We present the percent of trabecular bone volume BV/TV (bone volume / total volume), trabecular number (Tb.N), trabecular separation (Tb.Sp), and cortical cross‐sectional thickness (Cs.Th) in Fig. 1*D*. The BV/TV in the OVX group was significantly lower than in the sham or OVX plus E2 groups (*p* < 0.05); we found the BV/TV in the OVX plus E2 group was significantly higher than any other group (Fig. 1*D*). The differences in Tb.N among the three groups showed exactly the same pattern as we observed in changes in the BV/TV (Fig. 1*D*). The Tb.Sp in the OVX group was significantly higher than that in the sham or OVX plus E2 group; the OVX plus E2 group was the lowest of all three groups (Fig. 1*D*). A cortical parameter, Cs.Th showed the same pattern as the trabecular parameters BV/TV and Tb.N: They were all significantly lower in the OVX group, and these were significantly reversed by E2 treatment (OVX plus E2 group; Fig. 1*D*). Although μCT has higher resolution and more sensitivity for measuring bone structures such as BV and Tb.N, it does not measure BMD; therefore, we used pQCT as a confirmatory measure for BMD to evaluate bone quantity after ovariectomy and E2 replacement. Our data indicate that OVX‐associated alterations of both bone mass and structures in the tibia were reversed by the E2 treatment.

**Figure 2:**
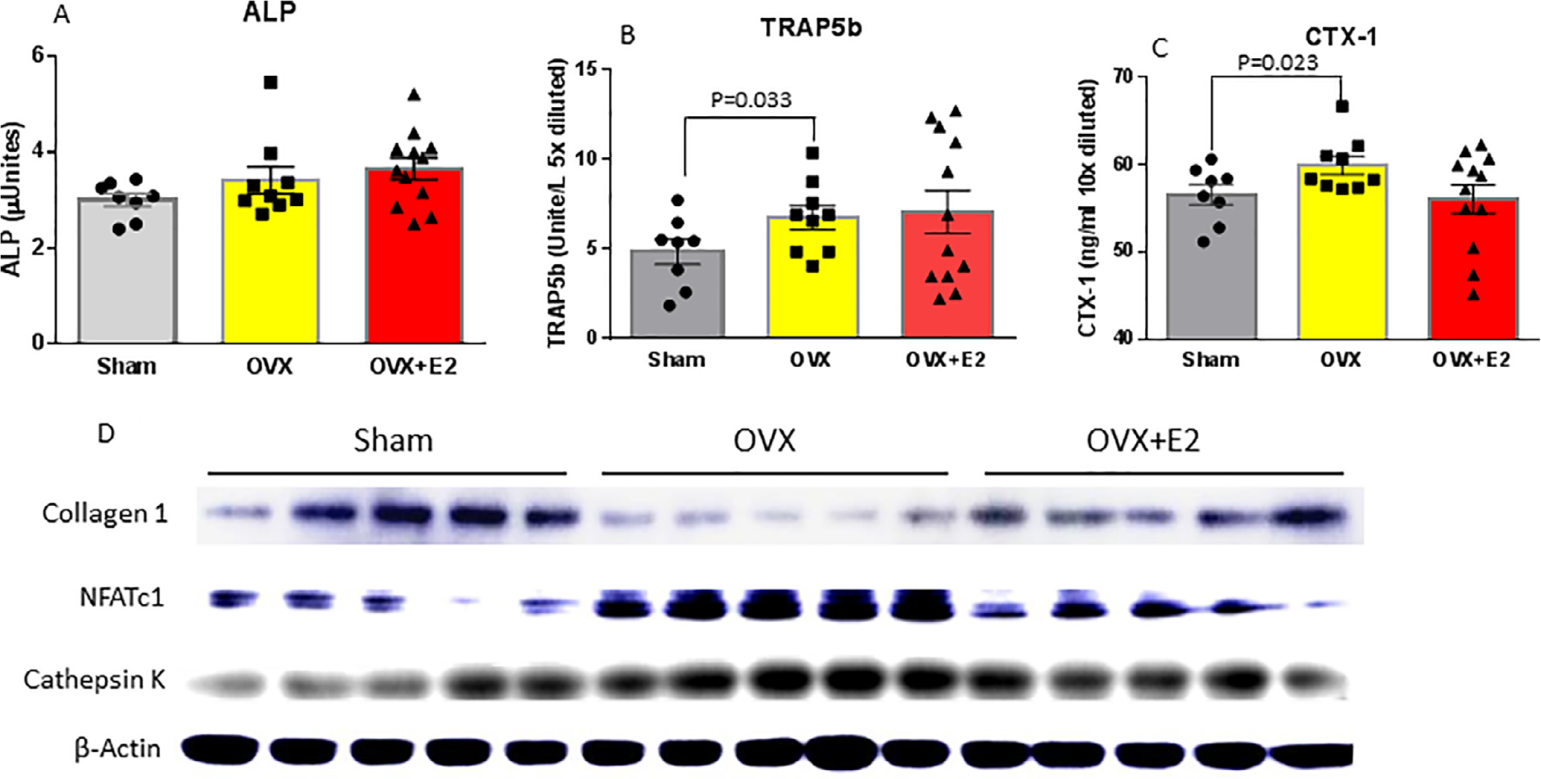
Serum bone turnover markers in three different treatment mouse groups. Standard ELISA methods for (*A*) APL, alkaline phosphatase; (*B*) TRAP5b, tartrate‐resistant acid phosphatase 5b; (*C*) CTX‐1, C‐terminal telopeptide of type I collagen. (*D*) Western blots for col‐1a (collagen 1), NFATc1, and cathepsin K. Total protein was isolated from vertebra L2 to L3; samples were five per group. Data are expressed as mean ± SD (sham group *n* = 9; OVX group *n* = 10; OVX + E2 *n* = 11). One‐way ANOVA followed by Student–Newman–Keuls post hoc analysis for multiple pairwise comparisons was performed.

Serum bone turnover markers were also measured. Because of limited amounts of serum, we were only able to measure three using ELISA (Fig. 2). In measuring ALP, we found that ALP was bone‐specific, but we did not see differences in all three groups (Fig. 2*A*). Interestingly, the bone resorption markers, TRAP5b reflecting osteoclast number (Fig. 2*B*) and CTX‐1 (Fig. 2*C*) reflecting osteoclast activity, were found to be significantly higher in the OVX mice compared with the sham mice. However, there were no statistical differences in the OVX plus the E2 mouse group compared with those either from the sham or OVX group. We isolated total protein from vertebra L4 from all groups of mice; samples were pooled to five mice per group. Collagen 1 protein expression was significantly decreased in the OVX group, but there were no differences between the sham and OVX plus E2 groups (Fig. 2*D*). OVX activated NFATc1 and cathepsin K protein expression; E2 ameliorated OVX effects on those protein expression (Fig. 2*D*).

### OVX activates senescence signaling and Nox4 expression in bone

**Figure 3:**
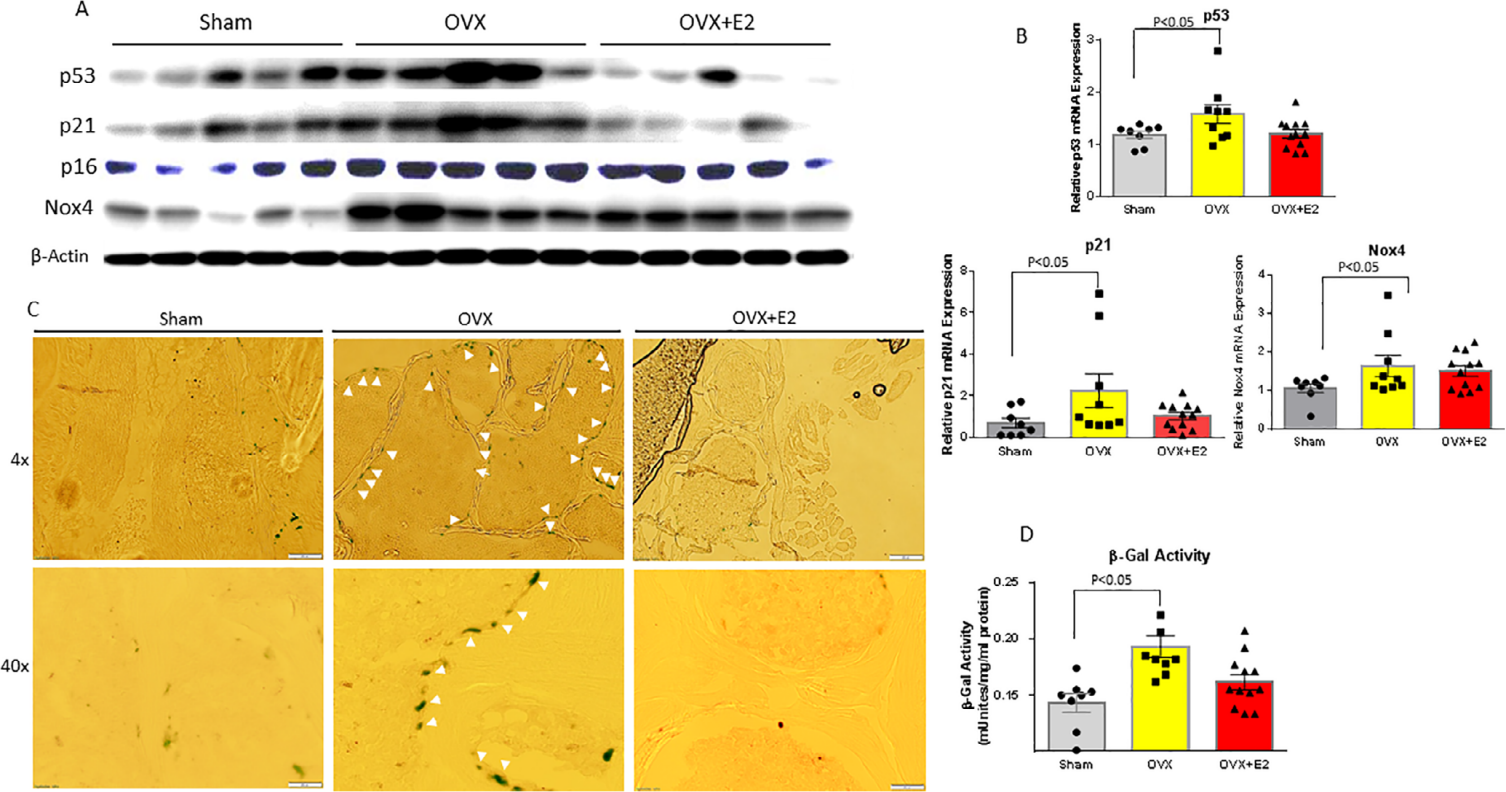
Increased senescence pathway in bone from ovariectomized (OVX) mice blocked by E2. (*A*) Proteins were isolated from L2 to L3 vertebral bone from mice in all three treatments; samples were pooled five per each group. Western blots show increased p53, p21, p16, and Nox4 expression by OVX; E2 treatment blocked OVX‐induced p53 and p21 protein expression, but not p16 and Nox4. (*B*) RNA was isolated from bone from all three treatment groups; real‐time PCR showed mRNA expression of p53, p21, and Nox4 similar patterns in their protein expression. (*C*) Senescence‐associated β‐galactosidase (SAβG) staining was performed on cryo‐sectioned L4 vertebral bone. Images at magnification ×4 and ×40 from epifluorescent microscope (model BH‐2; Olympus, Tokyo, Japan) show one representative sample from each group. White arrows show SaβG‐positive blue‐stained osteoblastic cells on bone surface. (*D*) SAβG (β‐gal) activity was measured (see Materials and Method section). Bar data are expressed as mean ± SD (sham group *n* = 9; OVX group *n* = 10; OVX + E2 *n* = 11). One‐way ANOVA followed by Student–Newman–Keuls post hoc analysis for multiple pairwise comparisons was performed.

We hypothesized that degenerative bone loss, including those induced by sex steroid deficiency, aging, and chronic alcohol consumption, is associated with increased bone osteoblast senescence.^(^ ^9^ ^)^ Previous research has suggested Nox proteins may be involved in osteoblast senescence signaling transduction.^(^ ^24^ ^)^ We measured p53, p21, and p16 senescence transduction molecules and Nox4 expression in bone from three different treatment groups. OVX increased p53, p21, p16, and Nox4 protein (Fig. 3*A*) and mRNA expression (Fig. 3*B*) compared with their expression in bone in sham mice. OVX plus E2 blunted OVX‐activated p53 and p21 in both protein (Fig. 3*A*) and mRNA expression (Fig. 3*B*), but not p16 protein expression at the levels seen in the sham mouse group. E2 did not significantly effect OVX‐induced Nox4 protein and mRNA expression (Fig. 3*A*, *B*). SAβG activity is a marker of aging or senescent cells; the SAβG activity in bone was measured in total proteins isolated from bone of all three groups. The SAβG activity in bone from the OVX group was significantly higher compared with those from the sham group. E2 treatment brought down the SAβG activity in bone from the OVX group to levels in the sham group (Fig. 3*D*). Moreover, using cryo‐sectioned vertebral bone samples and SAβG staining, we showed significantly increased numbers of clear blue‐stained SaβG‐positive cells on the surface of trabecular bone from the OVX group compared with samples from either the sham or E2 plus OVX groups (Fig. 3*C*). To identify if SAβG blue‐stained cells were osteoblastic cells, we performed triple‐costaining using Col‐1a and osteocalcin antibodies, in addition to SAβG staining (Supplementary Fig. S1). SABG blue‐stained cells expressed both Col‐1a and osteocalcin, two specific osteoblastic cell markers at very low levels. Senescent osteoblasts may be inactive and thus have limited expression of genes such as Col‐1a and osteocalcin related to osteoblast function. SAβG staining alone limits our ability to definitively identify these cells as osteoblasts. In this regard, we point out that we have previously identified p21/p53‐induced senescence in osteoblasts in vitro cultured in the presence of ethanol, which was also prevented by E2.^(^ ^9^ ^)^ These observations strongly suggest that OVX promotes osteoblast bone senescence, whereas E2 treatment prevents it, and that OVX‐associated induction of Nox4 may not be involved in this process.

### OVX induces bone loss and promotes bone osteoblast senescence in Nox4 gene‐deletion female mice

**Figure 4:**
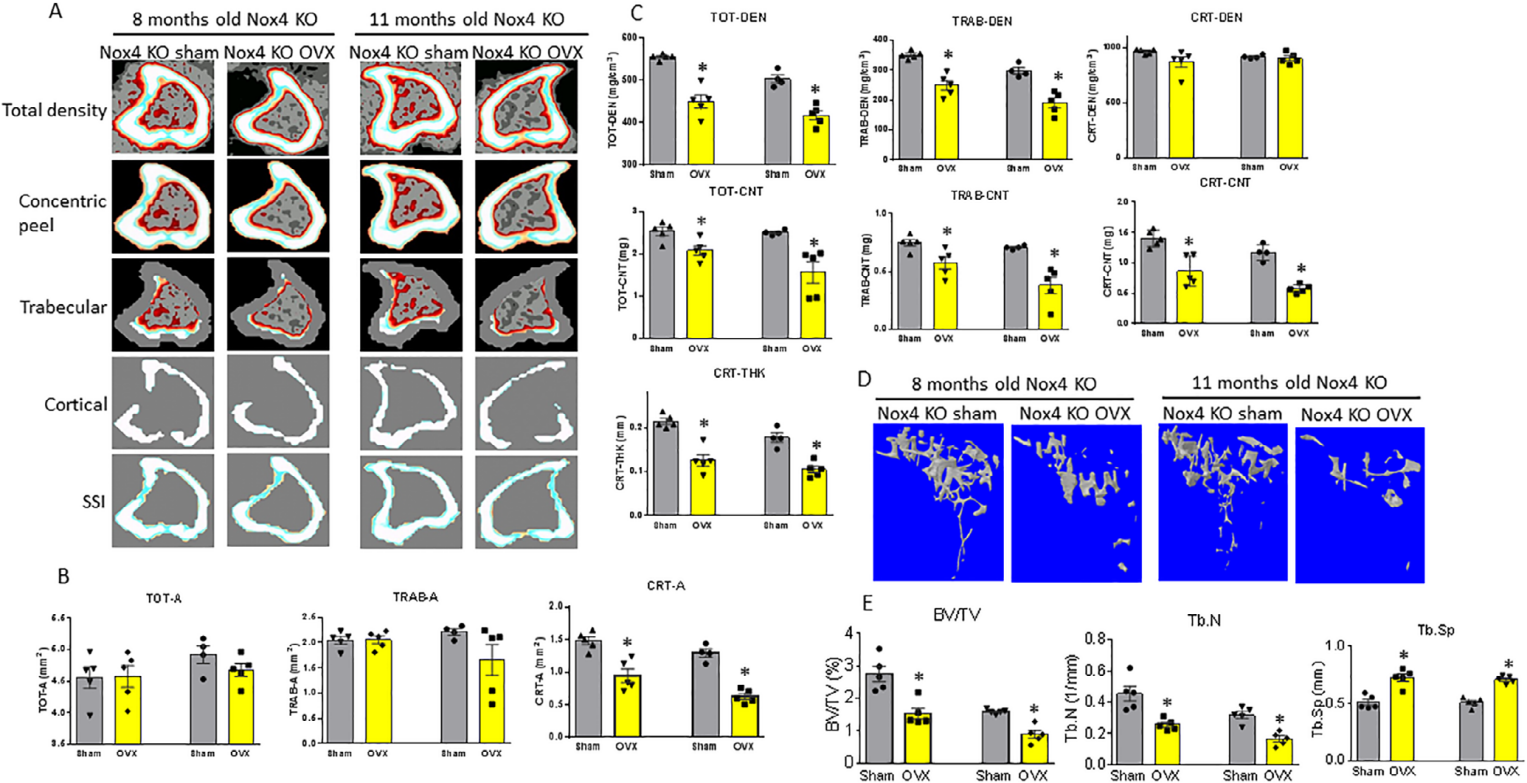
Ovariectomy induces bone loss in both 8‐ and 11‐month‐old Nox4^−/−^ mice. (*A*) Sagittal pQCT scan of one slice of the proximal tibia from one sample of each group of mice. Color changes from black to white indicate bone density from low to high. (*B*) pQCT measured tibia parameters from sham and ovariectomized (OVX) 8‐ and 11‐month‐old mouse groups. Left two bars are from 8‐month‐old sham and OVX mice, right two bars are from 11‐month‐old sham and OVX mice. TOT‐A, TRAB‐A, CRT‐A = total bone area, trabecular bone area, cortical bone area, mm^2^. (*C*) pQCT parameters continued. TOT‐DEN, TRAB‐DEN, CRT‐DEN = total BMD, trabecular BMD, cortical BMD, gm/cm^3^; TOT‐CNT, TRAB‐CNT, CRT‐CNT = total BMC, trabecular BMC, cortical BMC, mg; CRT‐THK = cortical thickness, mm. (*D*) Representative μCT images of the proximal tibia from one sample of each group of mice. (*E*) μCT measured three parameters from tibias from the sham and OVX 8‐ and 11‐month‐old mouse groups. BV/TV = bone volume/total tissue volume; Tb.N = trabecular number; Tb.Sp = trabecular separation. Data are expressed as mean ± SD (*n* = 5 per group). *p < 0.05 by *t* test between sham and OVX groups.

We next conducted experiments to determine if Nox4 gene deletion protects OVX‐induced bone loss and senescence in bone osteoblasts. We studied bone mass, structural changes, and senescence in the bones of mice with Nox4 whole‐body gene deletion after sham surgery or OVX mice at two different ages of 8 and 11 months. As shown in representative scans in Fig. 4*A*, by analyzing tibial bone mass parameters using pQCT, we found that there were no changes in total or trabecular bone areas (Fig. 4*B*). However, cortical bone area from OVX mice was significantly lower compared with those from respective sham mice at both 8 and 11 months of age (Fig. 4*B*). An analysis of BMD of each group, revealed that both total and trabecular BMD in mice from the OVX group were significantly reduced compared with sham groups for both 8‐ and 11‐month‐old mice (*p* < 0.05; Fig. 4*C*). Interestingly, despite the significant differences in cortical area between groups, there were no differences in cortical BMD between groups at both ages of the Nox4 gene‐deleted mice (Fig. 4*C*). BMC was also analyzed; total, trabecular, and cortical BMC were all lower in the OVX groups compared with those from the sham groups for both ages of Nox4 gene‐deleted mice (*p* < 0.05; Fig. 4*C*). This was also the case for cortical thickness (*p* < 0.05; Fig. 4*C*). The results in the Nox4^−/−^ mice in the OVX and sham groups were very similar to that described above in younger WT mice.

Structural changes of bone were also analyzed by μCT as shown in representative scans below the growth plate of the tibia in Fig. 4*D*. Percent of trabecular bone volume BV/TV, Tb.N, and Tb.Sp is presented in Fig. 4*E*. The BV/TV and Tb.N in the OVX groups were significantly lower than those from the sham groups at both ages (Fig. 4*E*). However, Tb.Sp in the OVX groups was higher than those from the sham groups at both ages of Nox4^−/−^ mice (*p* < 0.05; Fig. 4*E*).

**Figure 5:**
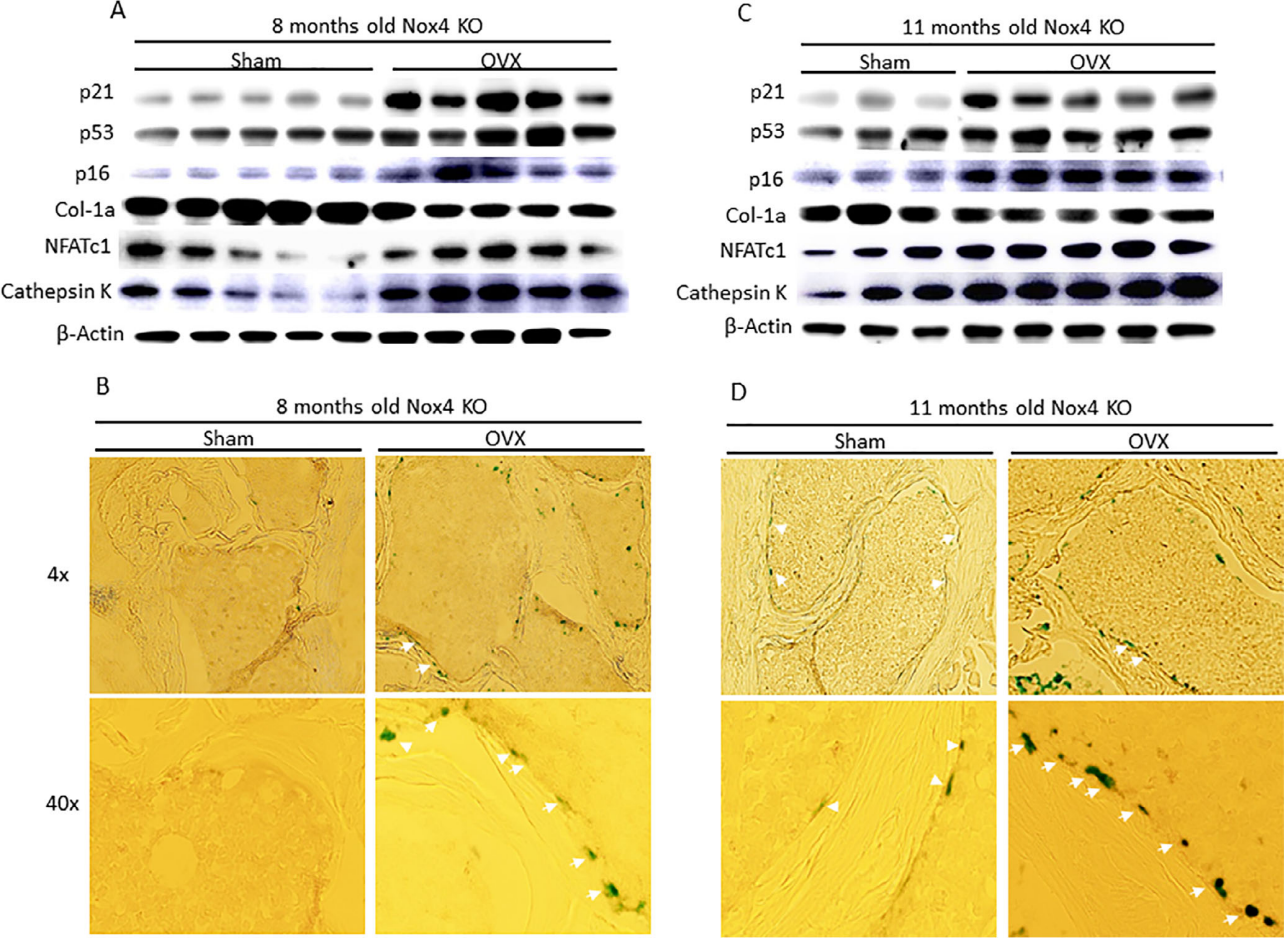
Increased senescence pathway in bone from ovariectomized (OVX) Nox4^−/−^ mice. (*A*) Proteins were isolated from L2 to L3 vertebral bone from sham and OVX 8‐month‐old mouse groups. Western blots show overexpression p53, p21, p16, NFATc1, and cathepsin K, but downregulation of Col‐1a protein expression in the OVX group compared with the sham group. (*B*) Senescence‐associated β‐galactosidase (SAβG) staining was performed on cryo‐sectioned L4 vertebral bone. Images (magnification ×4 and ×40 from an epifluorescent microscope, model BH‐2; Olympus, Tokyo, Japan) show one representative sample from each group. White arrows show SaβG‐positive blue‐stained osteoblastic cells on bone surface. (*C*) Western blots show overexpression of p53, p21, p16, NFATc1, and cathepsin K, but downregulation of Col‐1a protein expression in 11‐month‐old OVX group compared with the sham group (two samples from the sham group were lost during sample preparation). (*D*) SAβG staining was performed on cryo‐sectioned L4 vertebral bone. Images (magnification ×4 and ×40 from epifluorescent microscope, model BH‐2; Olympus) show one representative sample from each group of 11‐month‐old Nox4^−/−^ mice. White arrows show SaβG‐positive blue‐stained osteoblastic cells on bone surface.

We also measured the expression of p53, p21, and p16 senescence transduction proteins. Ovariectomy induced p53, p21, and p16 protein expression (Fig. 5*A*,*C*) compared with expression in bone from the sham mice in both ages of Nox4^−/−^ mice. Western blots were also performed for bone remodeling markers, including Col‐1a, NFATc1, and cathepsin K (Fig. 5*A*,*C*). Clearly, OVX in Nox4−/− mice activated senescence signaling and bone resorption markers, but reduced bone formation markers. Using cryo‐sectioned vertebral bone samples and SAβG staining, we showed significantly increased numbers of clear blue‐stained SaβG‐positive cells on the surface of trabecular bone from the OVX groups compared with samples from the sham groups at both ages of Nox4^−/−^ mice (Fig. 5*B*,*D*). Again, to identify whether the SAβG blue‐stained cells were true osteoblastic cells, we performed triple costaining using Col‐1a and osteocalcin antibodies, in addition to SaβG‐staining (Supplementary Fig. S2). However, SABG blue‐stained cells only expressed Col‐1a and osteocalcin two specific osteoblastic cell markers at low levels. These observations also strongly support the idea that OVX induces bone loss and promotes bone senescence, but that this process does not involve Nox4 induction.

### Changes in senescence‐associated gene expression in bone in response to OVX in both WT and Nox4^−/−^ mice

**Figure 6:**
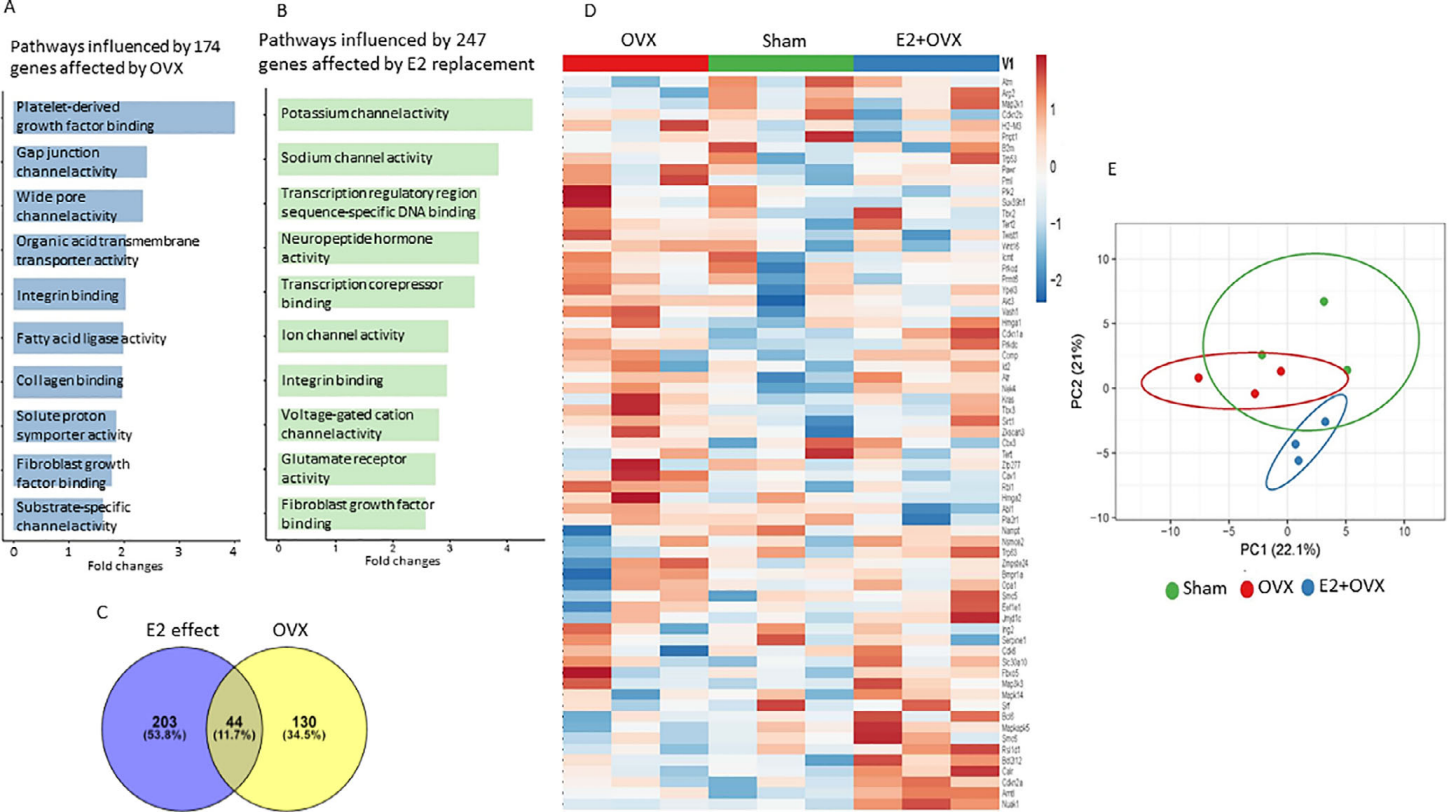
Pathway analysis of genes involved in bone senescence from RNA‐sequencing data of sham, OVX, and OVX plus E2 WT mice with at least a 1.5‐fold significant change in expression. RNA samples were pooled to three per each group for RNA‐Seq analysis. (*A*) Top‐10 overall enriched pathways using BiNGO gene ontology analysis from 174 genes that were at least 1.5‐fold significantly affected by OVX. (*B*) E2 treatment altered 247 genes from OVX mice; the top three were mostly involved in potassium channel activity, sodium channel activity, and transcription regulatory region sequence‐specific DNA binding. (*C*) Vine diagram of differential expression analysis of those significantly changed genes that were affected by E2 treatment and ovariectomy. (*D*) Heat map of top 67 genes related to senescence pathways representing three pools (OVX 1, 2, 3; sham 1, 2, 3; OVX + E2 1, 2, 3). (*E*) Principal component analysis of predicted functional metagenomics pathways of all senescence‐related genes using PICRUSt (Phylogenetic Investigation of Communities by Reconstruction of Unobserved States) (http://picrust.github.io/picrust/).

**Figure 7:**
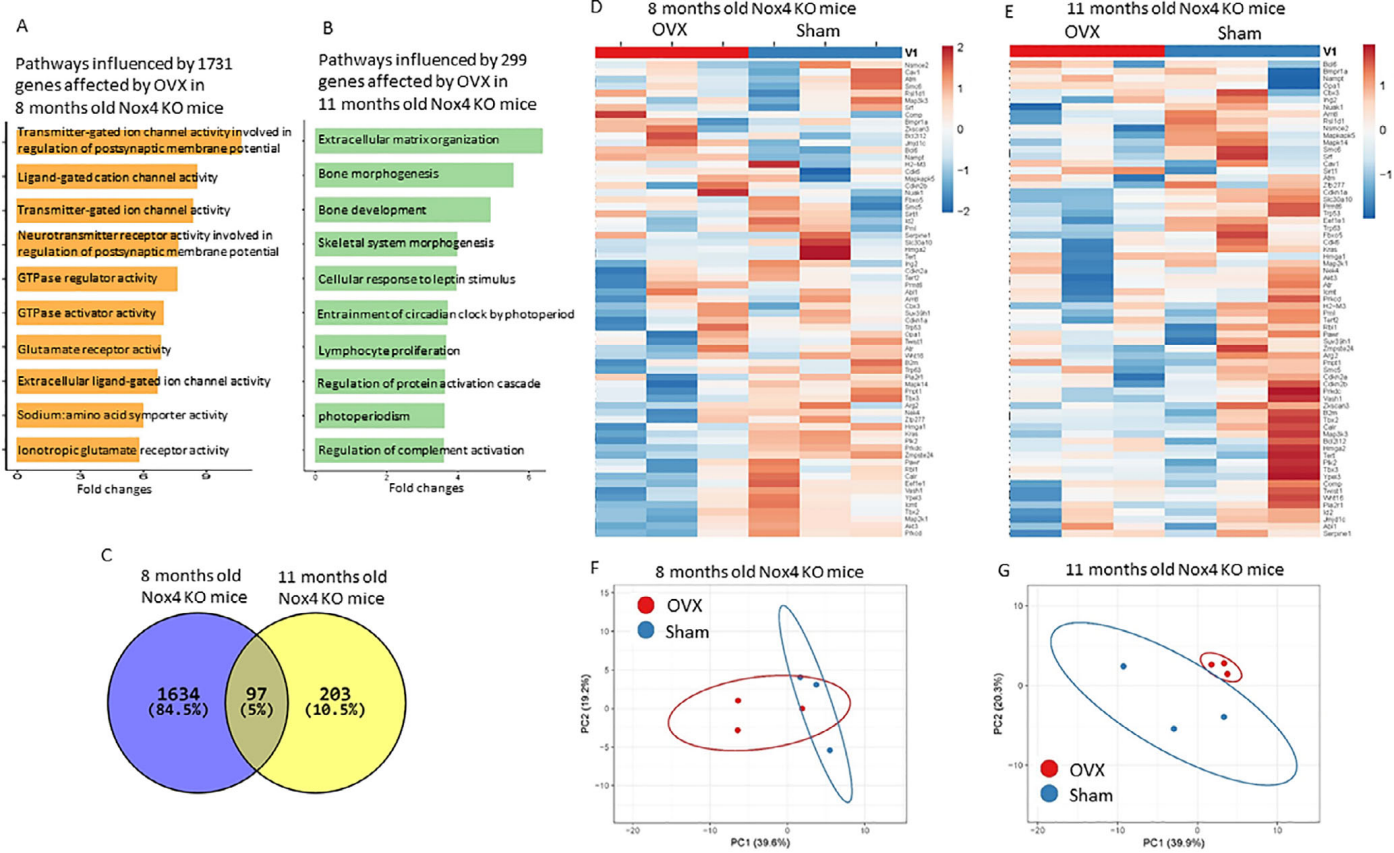
Pathway analysis of genes involved in bone senescence from RNA‐sequencing data of sham and ovariectomized (OVX) Nox4 gene‐deletion mice with at least a 1.5‐fold significant change in expression. RNA samples were pooled to three per each group for RNA‐seq analysis. (*A*) Top‐10 overall enriched pathways using BiNGO gene ontology analysis from 1731 genes that were at least 1.5‐fold significantly affected by OVX 8‐month‐old Nox4 gene‐deletion mice. (*B*) Top‐10 overall enriched pathways from 299 genes that were at least 1.5‐fold significant affected by OVX 11‐month‐old Nox4 gene‐deletion mice. (*C*) Vine diagram of differential expression analysis of those significantly changed genes that were affected by ovariectomy in differently aged Nox4 gene‐deletion mice. (*D*) Heat map of top 67 genes related to senescence pathways representing two pools (OVX 1, 2, 3; sham 1, 2, 3) from 8‐month‐old Nox4 gene‐deletion mice. (*E*) Heat map of top 67 genes related to senescence pathways representing two pools (OVX 1, 2, 3; Sham 1, 2, 3) from 11‐month‐old Nox4 gene‐deletion mice. (*F*) and (*G*) Principal component analysis of predicted functional metagenomics pathways of senescence‐related genes using PICRUSt (Phylogenetic Investigation of Communities by Reconstruction of Unobserved States) of samples from 8‐ and 11‐month‐old Nox4^−/−^ mice (http://picrust.github.io/picrust/).

To examine overall changes in gene expression in bone associated with OVX, E2 treatment, and Nox4 gene deletion at different ages, and to identify additional information on senescence‐associated genes in bone that are responsive to OVX‐induced bone loss in both WT and Nox4^−/−^ mice, we carried out RNA‐seq‐based gene expression profiling. As we found recently,^(^ ^31^ ^)^ bone‐specific genes collagen 1, osteocalcin [bone gamma‐carboxyglutamate (gla) protein], and osteonectin were among the top highly expressed genes based on RPKM values (normalized reads per kilobase of transcript per million mapped), indicating that the RNA preparations were, as expected, from enriched bone tissue. GO analysis using BiNGO (a biological network gene ontology tool) (https://www.psb.ugent.be/cbd/papers/BiNGO/Home.html) showed 174 genes that were at least 1.5‐fold significantly affected by OVX compared with sham (Fig. 6*A*). The genes included those involved in growth factor binding, integrin binding, collagen binding, and fibroblast growth factor binding (Fig. 6*A*). E2 treatment altered 247 genes from the OVX mice that were mostly involved in potassium channel activity, sodium channel activity, transcription regulatory region sequence‐specific DNA binding, integrin binding, and fibroblast growth factor binding (Fig. 6*B*). Differential expression analysis of the significantly changed genes showed that ovariectomy affected expression of 130 transcripts (±1.5‐fold change, *p* < 0.05), E2 affected expression of 203 transcripts (±1.5‐fold change, *p* < 0.05), and 44 transcripts (11.7%) had affected expression in common in OVX and E2 mice (Fig. 6*C*). We analyzed the genes that were involved in “senescence” as shown in the heat map presented in Fig. 6*D*. Principal component analysis of gene expression profiles showed that this set of transcripts discriminated the sham, OVX, and OVX + E2 samples, indicating significant effects of ovariectomy and E2 on senescence gene expression profiles in bone (Fig. 6*E*). We also analyzed RNA‐seq‐based gene expression profiling in the Nox4^−/−^, sham, and OVX mice. In 8‐month‐old Nox4^−/−^ mice, GO analysis showed 1731 genes that were significantly affected in OVX compared with sham mice (Fig. 7*A*). The genes involved in transmitter‐gated ion channel activity, the regulation of postsynaptic membrane potential, ligand‐gated cation channel activity, and transmitter‐gated ion channel activity, were mostly and significantly influenced in OVX mice (Fig. 7*A*). In 11‐month‐old Nox4^−/−^ mice, GO analysis found 299 genes that were significantly affected in OVX compared with sham mice (Fig. 7*B*). Interestingly, differential expression analysis of the significantly changed genes showed that ovariectomy affected expression of 1634 transcripts (±1.5‐fold change, *p* < 0.05) in 8‐month‐old Nox4^−/−^ mice, ovariectomy affected expression of 203 transcripts (±1.5‐fold change, *p* < 0.05) in 11‐month‐old Nox4^−/−^ mice, and 97 transcripts (5%) were affected in common in OVX mice at both ages (Fig. 7*C*). Moreover, ovariectomy in 11‐month‐old Nox4^−/−^ mice not only altered a substantial number of genes, 1731 versus 299, the gene expression profile also was different from what we found in OVX Nox4^−/−^ 8‐month‐old mice. The genes involved in extracellular matrix organization, bone morphogenesis, and bone development, which seem to be more bone‐specific, were significantly influenced by ovariectomy in 11‐month‐old Nox4^−/−^ mice (Fig. 7*B*). Of the genes known to be involved in senescence, the top 67 genes are shown in the heat map in Fig. 7*D* from 8‐month‐old Nox4^−/−^ mice, and in Fig. 7*E* from 11‐month‐old Nox4^−/−^ mice. Principal component analysis of gene expression profiles showed that the set the of senescence‐associated gene transcripts discriminated the sham and OVX samples, indicating significant effects of ovariectomy on senescence gene expression profiles in bone in both 8‐ and 11‐month‐old Nox4^−/−^ mice (Fig. 7*F*,*G*). These analyses are also consistent with the idea that Nox4 gene expression in bone may be important for many physiologic processes, but it may not be significantly involved in OVX‐induced senescence pathways.

## Discussion

In the present study, we investigated bone metabolism and osteoblast senescence signaling in OVX WT and Nox4^−/−^ mice. We have shown that OVX‐induced bone loss was significantly associated with increased senescence signaling. Nox4 expression was induced in bone from OVX mice; however, its induction is apparently not required for OVX‐induced bone loss or increased senescence signaling. In Nox4^−/−^ mice, regardless of age, significant bone loss and increased senescence pathways after OVX compared with those of respective age‐matched sham controls. OVX‐induced bone loss and increased senescence signaling were found to be Nox4‐independent. Recent evidence suggests that Nox‐dependent ROS signaling may play an important role in cell cycle progression and proliferation.^(^ ^34^, ^35^ ^)^ We have previously reported increased bone formation during development in p47^phox^ (an essential cofactor for Nox2 activation) KO mice that was reversed in old p47^phox^ mice compared with their respective WT controls.^(^ ^24^ ^)^ Together with the data presented in our current study, we believe that Nox‐induced ROS signaling in osteoblasts may be Nox subtype‐dependent to exert pathophysiologic effects in bone.

There is a growing body of evidence from previous mechanistic research on OVX‐ and aging‐induced bone loss, suggesting that the function or life span of osteoblasts is decreased, whereas the activity and numbers of osteoclasts are increased. Estrogen replacement is able to prevent OVX‐induced bone loss, presumably via inhibiting osteoblast apoptosis, but promoting osteoclast apoptosis.^(^ ^36^, ^37^ ^)^ Other possible mechanisms of action of estrogen on bone cells are thought to be mediated via nitric oxide^(^ ^38^, ^39^, ^40^ ^)^ to suppress oxidative stress. However, much more research is still needed to differentiate the effects of estrogen on bone cells from other estrogen‐sensitive tissues. The therapeutic challenge is to keep estrogen effects on bone cells, but to avoid its unwanted effects on reproductive tissue proliferation.^(^ ^41^ ^)^ In our current report, we performed OVX experiments in both WT and Nox4^−/−^ mice. Our data demonstrate that bone formation and osteoblast function were both decreased; this was accompanied by increased bone resorption, indicating unbalanced bone remodeling. These findings are very similar to those observed in the skeleton of older Nox4 KO mice compared with younger mice. Unbalanced bone remodeling appeared to be associated with increased senescence in bone osteoblastic cells. We found clearly increased SAβG activity, and p53, p21, and p16 induction. Using a modified bone histologic method, we identified senescent SaβG‐positive osteoblastic cells on the surface of bone, although there are limitations to definitively identifying the specific lineage of senescent cells using only SaβG‐staining. This was the clearest evidence of an association between increased bone cell senescence and degenerative bone loss.^(^ ^24^ ^)^ Induction of Nox4 expression and activity appears to have only a minimal role in osteoblastic cell senescence, but increased osteoblastic cell senescence did appear to be p53‐dependent^(^ ^15^ ^)^ and reversed by E2 treatment consistent with our previous in vitro data on ROS‐driven osteoblast senescence mediated via ethanol exposure.^(^ ^9^ ^)^

Increased oxidative stress or accumulation of ROS in bone or bone marrow is believed to be one of the key mechanisms that explains accelerated bone degeneration after sex steroid loss or aging. ROS can be generated by several sources; activation of NADPH‐oxidases represents one of the major sources of ROS signaling molecules, including superoxide, hydrogen peroxide, and hydroxyl radicals in many cell types. Activation mechanisms, tissue distribution, and subcellular localization of different members of the Nox family are markedly different, and their expression patterns are also cell‐specific. In bone, osteoclasts express all four types of Nox (Nox1 to 4), whereas we have previously identified that osteoblasts do not express Nox3, but do express Nox1, Nox2, and Nox4. Nox2 and Nox4 were both highly expressed in osteoblastic cells.^(^ ^23^ ^)^ Although we do not know the mechanisms by which specific activation of each Nox subtype occurs, Nox expression in different cell types may have distinguished roles, which they usually coordinate to generate ROS. We have previously shown that activation of Nox2 and its signaling in bone was significantly reduced in p47^phox−/−^ mice. p47^phox^ is a subunit of Nox2 required for its activation.^(^ ^42^, ^43^ ^)^ We observed increased bone formation during development in early life of p47^phox−/−^ mice that was reversed in old p47^phox−/−^ mice compared with their respective WT controls.^(^ ^24^ ^)^ Therefore, we concluded that Nox2‐mediated reduction of ROS generation appears only temporally beneficial for early bone development. This may also be the case in Nox4^−/−^ mice. In our current report, we found that Nox4 was induced in OVX mice. However, bone loss after OVX in Nox4^−/−^ mice was actually greater compared with that in WT mice: 17% versus 14% in total bone BMD, 36% versus 23% in trabecular BMD. These numbers were calculated from the pQCT data presented in Figs. 1 and 4, total BMD 505 ± 34 mg/cm^3^ in WT shams versus 436 ± 42 mg/cm^3^ in WT OVX; trabecular BMD 292 ± 29 mg/cm^3^ in WT sham versus 223 ± 40 mg/cm^3^ in WT OVX; total BMD 502 ± 19 mg/cm^3^ in Nox4 KO sham versus 417 ± 24 mg/cm^3^ in Nox4 KO OVX; trabecular BMD 297 ± 22 mg/cm^3^ in Nox4^−/−^ sham versus 191 ± 36 mg/cm^3^ in Nox4^−/−^ OVX. Although Nox4 KO mice were 3 weeks older than WT mice, all these findings are consistent with μCT data and suggest that Nox4 expression may help to maintain bone remodeling in the adult. Taken together with our previous observations,^(^ ^24^, ^44^ ^)^ using whole‐body Nox4 or Nox2 gene deletion mouse models to determine whether Nox plays a direct role in intact animals during bone development seems difficult. To examine the direct role of Nox4 on bone development and remodeling, we are currently generating bone cell‐type‐specific Nox4 or Nox2 gene‐deletion mouse models to answer the specific question of whether Nox4 or Nox2 plays a role in osteoblast differentiation and proliferation at different ages.^(^ ^45^ ^)^

We have previously hypothesized that accumulated ROS might be the toxic byproduct of cellular function. However, specific Nox‐dependent ROS signaling may actually be required for normal cellular functions.^(^ ^46^, ^47^ ^)^ Because of limited samples, we were unable to measure ROS levels in bone tissue in our current study. We suspect that under normal conditions, for small amounts of ROS produced by Nox enzymes in particular, cellular compartments are necessary for the physiologic functions of cells, but not sufficient to induce pathologic actions. Therefore, reduced ROS production caused by any type of Nox gene deletion was not able to compensate OVX‐induced senescence in bone. Although cellular apoptosis is important, we believe that accelerated senescence in osteoblasts is pivotal for OVX‐ and aging‐induced bone loss. Osteoblast differentiation and proliferation are unique processes that are dependent on the status of the cell senescence signaling pathway, ie, increased signaling through the senescence pathway restricts the capacity of a cell to proliferate and its ability to differentiate.^(^ ^15^ ^)^ This idea is not only proven by showing senescent osteoblast and increased SAβG activities in bone, but also fully supported by our RNA‐seq results. We have particularly analyzed genes involved in cell senescence pathways. Among total senescence‐associated genes that are affected by OVX, about 11.7% were reversed by E2 treatment. Moreover, an additional 5% of senescent genes from relatively younger Nox4 KOs were further activated when they became older. Additional studies are required to examine the details of increased oxidative stress and its association with osteoblast senescence and degenerative bone loss, as well as the critical biological targets in aging bone.

In conclusion, by using both WT and Nox4^−/−^ mouse models, we have shown that both OVX‐induced bone loss and decreased bone mass in older mice were significantly associated with increased osteoblast senescence signaling. OVX‐induced bone loss and increased osteoblast senescence signaling appear Nox4‐independent. Our data indicate that Nox4 plays a minimal role during skeletal involution, but it may have unique functions during bone development and on osteoblast differentiation and proliferation.

## Disclosures

The authors have declared that they have no conflict of interest.

## Supporting information


**Supplementary Figure S1.** identification of senescent osteoblbastic cells in bone. Triple staining of senescence‐associated 13‐Galactosidase (SA(3G) staining (blue), collagen lA (Col la) antibody immune staining (green) and osteocalcin antibody immune staining (red) were performed on cryosentioned L4 vertebrae bone, DAN stains for nucleus. Images of magnification 4x from epifluorescent microscope (model SH‐2, Olympus) showing one represented sample from each group. White arrows show blue SAPG positively stained osteoblastic cells on bone surface.

**Supplementary Figure S2.** identification of senescent osteoblbastic cells in bone. Triple staining of senescence‐associated 13‐Galactosidase (SAI3G) staining (blue), collagen 1A1 (Col lal) antibody immune staining (green) and osteocalcin antibody immune staining (red) were performed on cryosentioned L4 vertebrae bone. DAN stains for nucleus. Images of magnification 4x from epilluoresc ent microscope (model SH‐2, Olympus) showing one represented sample from each group. White arrows show blue SAI3G positively stained osteoblastic cells on bone surface.
