# Supplementary figures and images for: Nox4 Expression Is Not Required for OVX‐Induced Osteoblast Senescence and Bone Loss in Mice
Source: JBMR Plus. 2020 Jul 23;4(8):e10376. doi: 10.1002/jbm4.10376 (PMC7422714; doi:10.1002/jbm4.10376)

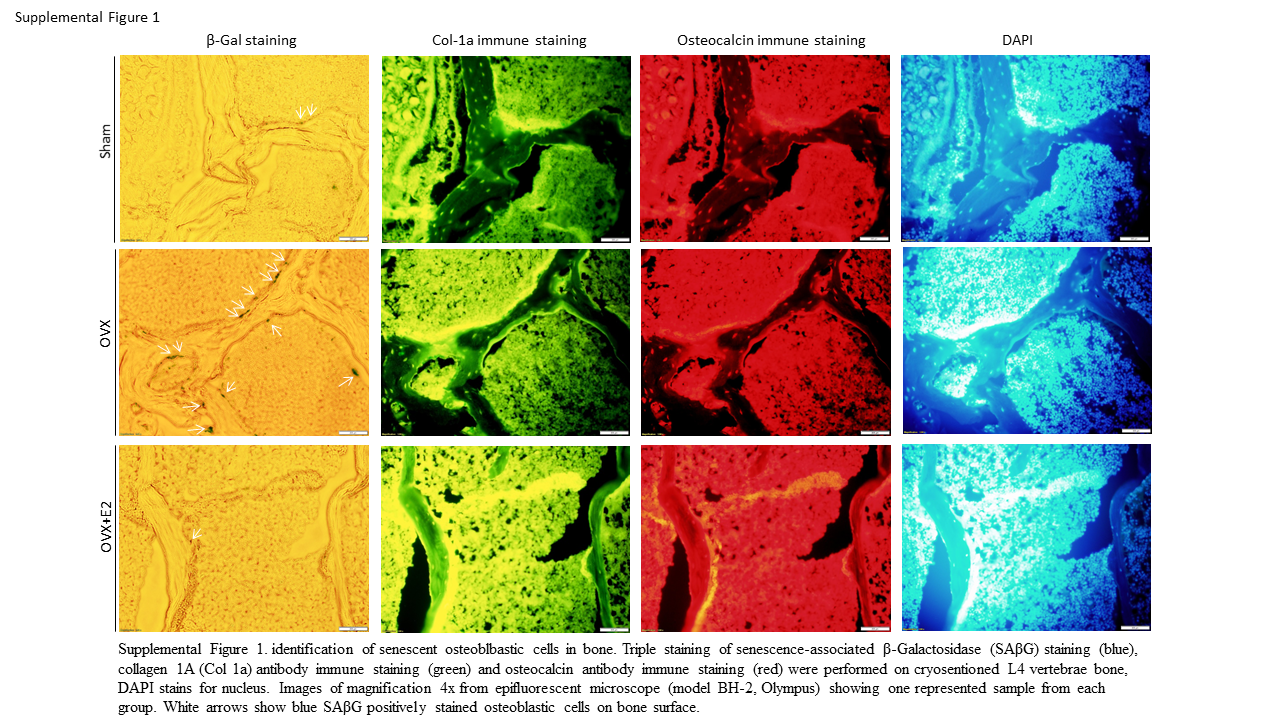

Supplement: Supplementary file 1 — Supplementary Figure S1. identification of senescent osteoblbastic cells in bone. Triple staining of senescence‐associated 13‐Galactosidase (SA(3G) staining (blue), collagen lA (Col la) antibody immune staining (green) and osteocalcin antibody immune staining (red) were performed on cryosentioned L4 vertebrae bone, DAN stains for nucleus. Images of magnification 4x from epifluorescent microscope (model SH‐2, Olympus) showing one represented sample from each group. White arrows show blue SAPG positively stained osteoblastic cells on bone surface. [file JBM4-4-e10376-s001.tif]

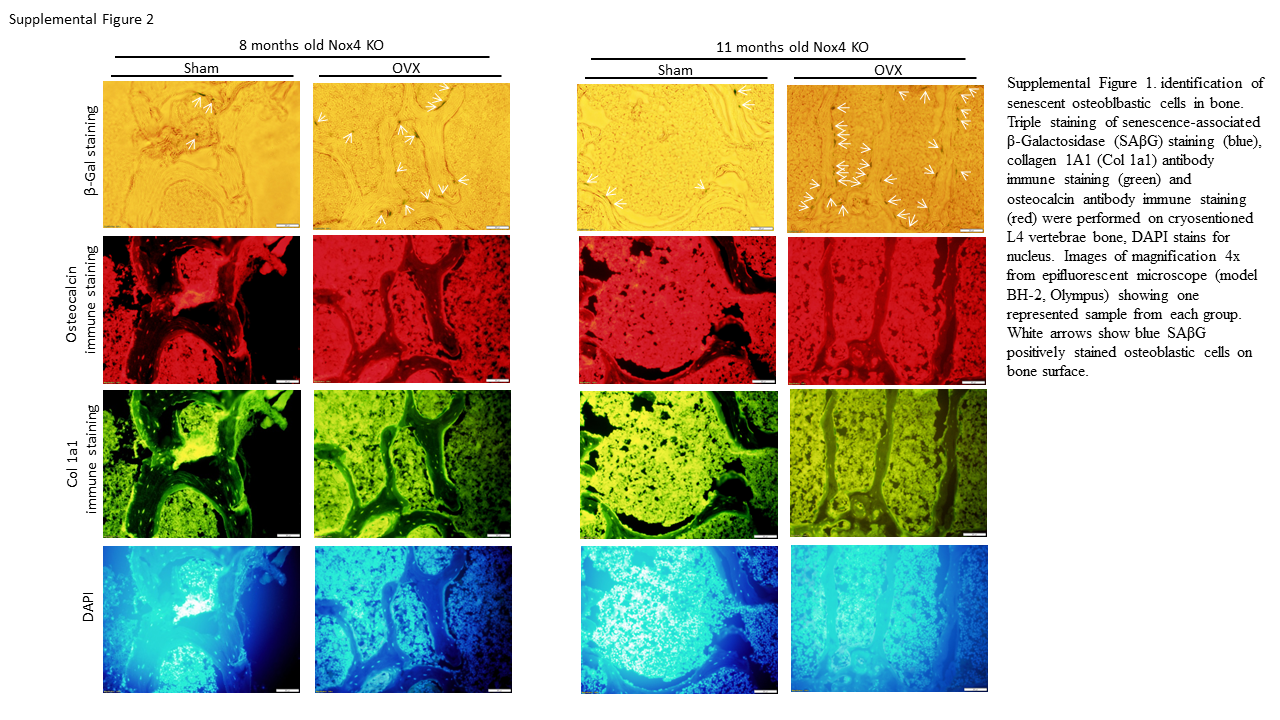

Supplement: Supplementary file 2 — Supplementary Figure S2. identification of senescent osteoblbastic cells in bone. Triple staining of senescence‐associated 13‐Galactosidase (SAI3G) staining (blue), collagen 1A1 (Col lal) antibody immune staining (green) and osteocalcin antibody immune staining (red) were performed on cryosentioned L4 vertebrae bone. DAN stains for nucleus. Images of magnification 4x from epilluoresc ent microscope (model SH‐2, Olympus) showing one represented sample from each group. White arrows show blue SAI3G positively stained osteoblastic cells on bone surface. [file JBM4-4-e10376-s002.tif]
